# Carnitine palmitoyltransferase gene upregulation by linoleic acid induces CD4^+^ T cell apoptosis promoting HCC development

**DOI:** 10.1038/s41419-018-0687-6

**Published:** 2018-05-23

**Authors:** Zachary J. Brown, Qiong Fu, Chi Ma, Michael Kruhlak, Haibo Zhang, Ji Luo, Bernd Heinrich, Su Jong Yu, Qianfei Zhang, Andrew Wilson, Zhen-Dan Shi, Rolf Swenson, Tim F. Greten

**Affiliations:** 1 Gastrointestinal Malignancy Section, Thoracic and Gastrointestinal Oncology Branch, Center for Cancer Research, National Cancer Institute, National Institutes of Health, Bethesda, MD 20892 USA; 2 Experimental Immunology Branch, Center for Cancer Research, National Cancer Institute, National Institutes of Health, Bethesda, MD 20892 USA; 3 Laboratory of Cancer Biology and Genetics, Center for Cancer Research, National Cancer Institute, National Institutes of Health, Bethesda, MD 20892 USA; 4 Department of Internal Medicine and Liver Research Institute, Seoul National University College of Medicine, Seoul, Korea; 5 Imaging Probe Development Center, National Heart, Lung, and Blood Institute, National Institutes of Health, Bethesda, MD 20892 USA

## Abstract

Hepatocellular carcinoma (HCC) is a common cause of cancer-related death worldwide. As obesity and diabetes become more prevalent, the contribution of non-alcoholic fatty liver disease (NAFLD) to HCC is rising. Recently, we reported intrahepatic CD4^+^ T cells are critical for anti-tumor surveillance in NAFLD. Lipid accumulation in the liver is the hallmark of NAFLD, which may perturb T cell function. We sought to investigate how the lipid-rich liver environment influences CD4^+^ T cells by focusing on carnitine palmitoyltransferase (CPT) family members, which control the mitochondrial β-oxidation of fatty acids and act as key molecules in lipid catabolism. Linoleic acid (C18:2) co-localized within the mitochondria along with a corresponding increase in CPT gene upregulation. This CPT upregulation can be recapitulated by feeding mice with a high-C18:2 diet or the NAFLD promoting methionine-choline-deficient (MCD) diet. Using an agonist and antagonist, the induction of CPT genes was found to be mediated by peroxisome proliferator-activated receptor alpha (PPAR-α). CPT gene upregulation increased mitochondrial reactive oxygen species (ROS) and led to cell apoptosis. In vivo, using liver-specific inducible MYC transgenic mice fed MCD diet, blocking CPT with the pharmacological inhibitor perhexiline decreased apoptosis of intrahepatic CD4^+^ T cells and inhibited HCC tumor formation. These results provide useful information for potentially targeting the CPT family to rescue intrahepatic CD4^+^ T cells and to aid immunotherapy for NAFLD-promoted HCC.

## Introduction

Hepatocellular carcinoma (HCC) is the most common primary liver cancer and the fourth leading cause of cancer-related death worldwide^1, 2^. HCC often arises in patients with liver cirrhosis caused by chronic hepatitis B or C virus infection. However, recent epidemiology studies found that non-alcoholic fatty liver disease (NAFLD) is also a high-risk factor for HCC^3^. NAFLD and its advanced form, non-alcoholic steatohepatitis (NASH), are recognized as the liver disease associated with metabolic syndrome and characterized by increased fat deposition in the hepatocytes. The prevalence of NAFLD is increasing rapidly with the growing epidemics of diabetes and obesity, and is thought to be present in up to one-third of the general population^4, 5^. Furthermore, it was estimated in 2012 that one in four liver cancers worldwide were attributable to diabetes and high BMI^6^. NAFLD is becoming a serious public health issue; however, there is no effective treatment so far, and the mechanism of how NAFLD promotes HCC development is still largely unknown.

There is accumulating data suggesting that metabolic changes in the tumor microenvironment may change immune metabolism and thereby promote or impair anti-tumor immunity^7^. Our recent study demonstrated that under NAFLD conditions, increased liver linoleic acid (C18:2), but not palmitic acid (C16:0), changes the metabolism of intrahepatic CD4^+^ T cells and leads to apoptosis, which contributes to HCC development^8^. The anti-tumor functions of CD4^+^ T cells in different types of cancer including liver cancer are starting to be recognized^9^. Using a murine HCC model induced by diethylnitrosamine (DEN), CD4^+^ T cells have been found to prevent tumor initiation and mediate the clearance of premalignant hepatocytes^10^. In humans, adoptive transfer of tumor-specific CD4^+^ T cells caused a complete tumor eradication in a patient bearing cholangiocarcinoma, another primary liver cancer^11^. Furthermore, immunotherapy is becoming standard of care for the treatment of advanced HCC. Nivolumab, an anti-PD-1 immune checkpoint inhibitor, has recently been approved by the United States Food and Drug Administration for the treatment of advanced HCC patients who have progressed on sorafenib^12^. Since NAFLD affects intrahepatic CD4^+^ T cells, the question of how NAFLD influences the efficacy of immunotherapy for liver cancer needs to be evaluated. To address this question, a better understanding of the influences of fatty liver environment on T cell metabolism is required. This may also shed light on the design of a targeted therapy and potentially a combined immunotherapy for HCC.

The carnitine palmitoyltransferase (CPT) system is responsible for transporting long-chain fatty acids from the cytoplasm into the mitochondria where the fatty acids undergo β-oxidation. This CPT system contains two separate proteins localized in the outer (CPT1) and the inner (CPT2) mitochondrial membrane^13^. While CPT2 is ubiquitously expressed, there are three tissue-specific CPT1 isoforms: CPT1a, CPT1b, and CPT1c^13^. CPT1a is the primary isoform in lymphocytes, liver, kidney, spleen, lung, intestine, pancreas, and ovary. CPT1b is highly expressed in skeletal muscle, heart, and adipose tissue, while CPT1c is predominately expressed in the brain^13^. Although our previous in vitro study showed C18:2 mediates CPT1a induction, the details of how the CPT genes are regulated in CD4^+^ T cells in the context of NAFLD and their potential role in HCC development are still unknown^8^.

The peroxisome proliferator-activated receptors (PPARs) are a group of lipid receptors and lipid-activated transcription factors that control metabolism and energy homeostasis. They can be divided into three subtypes: PPAR-α, PPAR-γ, and PPAR-β/δ. Among these, both PPAR-α and PPAR-γ are expressed in lymphocytes. PPAR-α is the main isoform expressed in the liver, with a much higher binding affinity for many fatty acids, including C18:2, compared to PPAR-γ or PPAR-β/δ^14^. It has been shown CPT1 is markedly induced by fatty acids which activate PPAR-α^15^. There are also studies showing PPAR-α, together with peroxisome proliferator-activated receptor gamma coactivator (PGC-1), upregulate the transcription of the CPT1a gene directly^16, 17^. Additionally, it has been demonstrated there is an increased level of PPAR-α mRNA in human HCC^18^. We hypothesized that the upregulation of the CPT genes was through PPAR-α, and by inhibiting PPAR-α we may reduce CPT induction and subsequent CD4^+^ T cell apoptosis when exposed to fatty acids.

In this study, we investigated the changes of CD4^+^ T cells in NAFLD focusing on CPT genes. C18:2 co-localized within the mitochondria with a corresponding increase in CPT gene upregulation. This increase can be repeated in intrahepatic CD4^+^ T cells by feeding mice with a C18:2-rich diet or NAFLD-inducing diet. Further studies demonstrated that PPAR-α mediates the upregulation of CPT genes. Induction of CPT genes increased reactive oxygen species (ROS) and led to CD4^+^ T cell apoptosis. In vivo treatment of mice with perhexiline, a CPT inhibitor, decreased apoptosis of intrahepatic CD4^+^ T cells and inhibited HCC development in NAFLD. These results provide useful information for targeting the CPT genes for NAFLD-promoted HCC therapy.

## Results

### Linoleic acid probes co-localize with the mitochondria

**Fig. 1:**
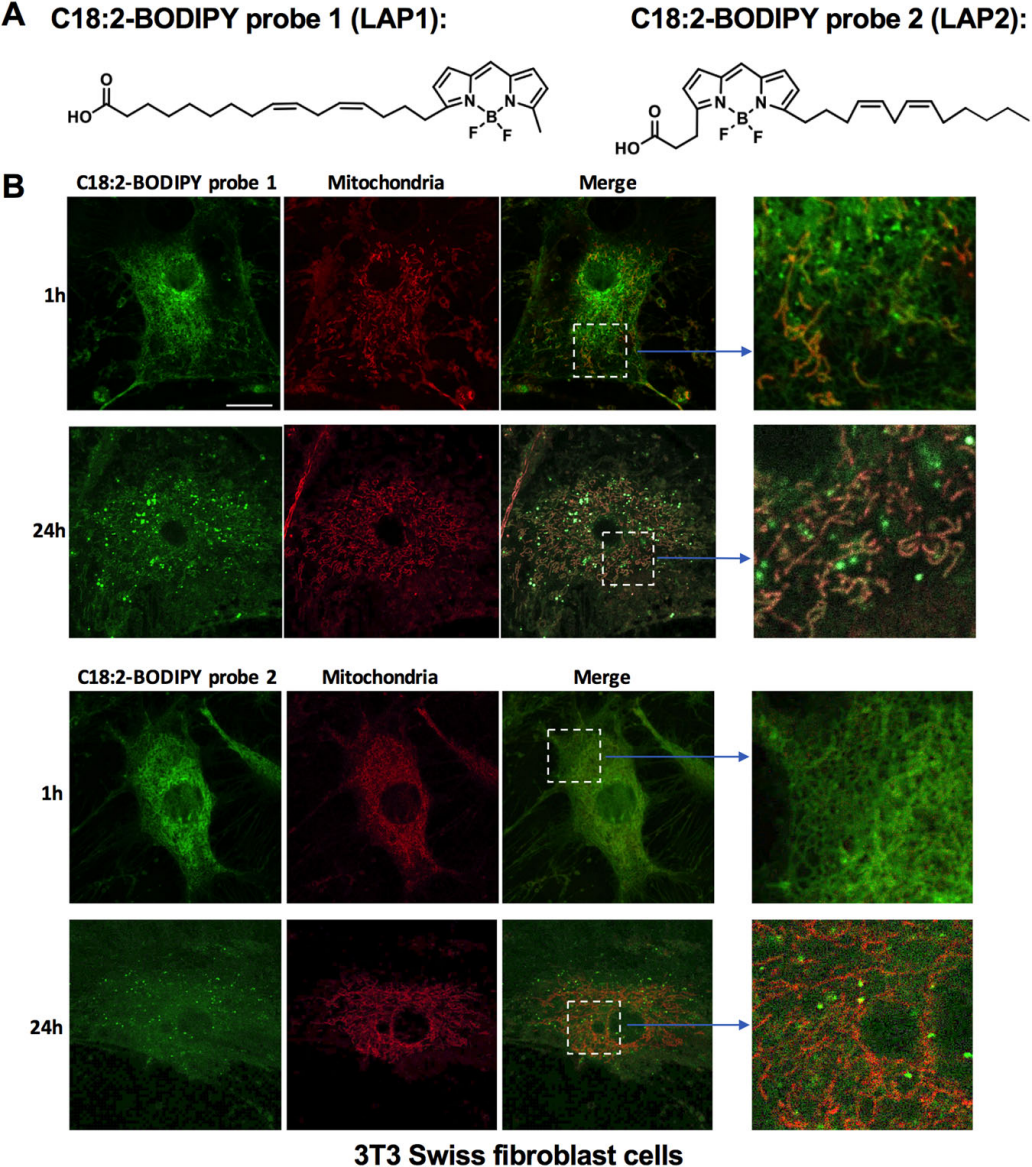
Linoleic acid probes co-localize with the mitochondria. **a** Schematic structure of C18:2-BODIPY probe 1 (LAP1) and probe 2 (LAP2). **b** Mouse 3T3 fibroblast cells were incubated with 2 µM C18:2-BODIPY Probe 1 or Probe 2 together with 10 nM MitoTracker Deep Red for 30 min. Live images were taken at 1 h or 24 h after staining. Scale bar: 20 µm

Our recent study found that intrahepatic CD4^+^ T cells are an indispensable component of anti-tumor surveillance in NAFLD, and linoleic acid (C18:2) causes CD4^+^ T cell apoptosis by impairing electron transport chain (ETC) function and generating ROS^8^. We therefore sought to further investigate the molecular mechanism of C18:2-induced apoptosis in the context of T cell metabolism. Long-chain fatty acids like C18:2 are transported into mitochondria by CPT gene members. During this process, CPT1 catalyzes the transfer of the acyl group of a long-chain fatty acyl-CoA from coenzyme A to carnitine so the resulting acylcarnitine can cross the mitochondrial outer membrane, while CPT2 reverses this reaction inside mitochondria^13^. In order to visualize mitochondrial uptake of the fatty acid C18:2, we synthesized two BODIPY fluorophore-conjugated linoleic acid probes (LAP1 and LAP2) to mimic C18:2 with similar sizes but differing in the position of the BODIPY fluorophore (Fig. 1a). Both probes displayed time-dependent co-localization with mitochondria in 3T3 cells (Fig. 1b) and Jurkat cells (Supplementary Fig. 1). There appeared to be more co-localization of LAP1 with mitochondria than LAP2 at 24 h time point, which may be attributable to different location of BODIPY fluorophore inside the probe.

### CPT genes in T cells are upregulated to different levels by C18:2 in vitro

**Fig. 2:**
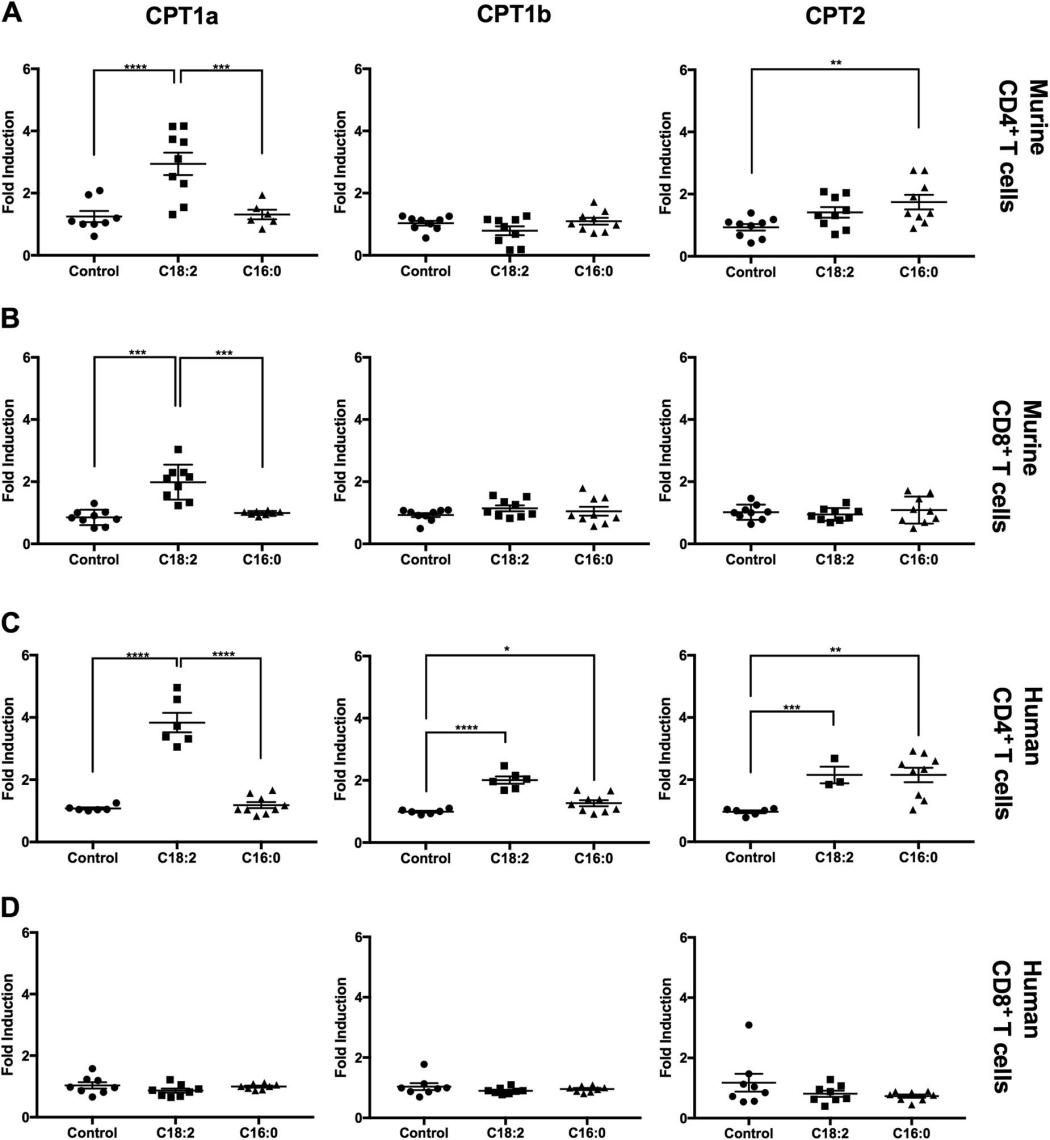
Linoleic acid upregulates CPT gene expression in murine and human lymphocytes. **a** Mouse splenic CD4^+^ T cells were isolated and treated with 100 µM fatty acids in vitro for 24 h. The CPT gene (CPT1a/CPT1b/CPT2) mRNA levels were measured by RT-qPCR. **b** Mouse splenic CD8^+^ T cells were isolated and treated as above with 100 µM fatty acids in vitro for 24 h. The CPT gene mRNA levels were measured by RT-qPCR. **c** Human CD4^+^ T cells were isolated from healthy donor peripheral blood mononuclear cells (PBMCs) and treated with 100 µM fatty acids in vitro for 24 h. The CPT gene mRNA levels were measured by RT-qPCR. **d** Human CD8^+^ T cells were isolated from healthy donor peripheral blood mononuclear cells (PBMCs) and treated with 100 µM fatty acids in vitro for 24 h. The CPT gene mRNA levels were measured by RT-qPCR. *P < 0.05, **P < 0.01, ***P < 0.001, ****P < 0.0001

Transport of C18:2 into the mitochondrial matrix requires both CPT1 and CPT2, with CPT1 being the rate limiting step^19^. Therefore, we sought to further test C18:2 induction of CPT genes by culturing T cells with C18:2 in vitro and measuring CPT mRNA expression. Murine CD4^+^ T cells were isolated from splenocytes of C57BL/6 mice and cultured with C18:2 or C16:0. C16:0 was used as a control as it was shown to not cause CPT1a upregulation in our previous study^8^. Murine CD4^+^ T cells cultured with C18:2 showed up to a 4-fold increase in CPT1a, as well as a 2-fold increase in CPT2 when cultured with C16:0 (Fig. 2a). Murine CD8^+^ T cells were treated in a similar manner, and showed an induction of CPT1a to approximately 2-fold, less than that observed in CD4^+^ T cells (Fig. 2b).

We also tested human CD4^+^ and CD8^+^ T cells from healthy donors in the same way. The expression level of all three CPT genes in CD4^+^ T cells increased after C18:2 treatment with the greatest induction seen in CPT1a (4-fold) (Fig. 2c). The expression of CPT1b and CPT2, but not CPT1a was upregulated by C16:0, suggesting that other lipids may also contribute to the induction of CPT genes in NAFLD. We did not observe induction of CPT genes in human CD8^+^ T cells (Fig. 2d). Thus, our results show that C18:2 can significantly increase the expression of CPT1a in both murine and human CD4^+^ T cells. Of note, these results cannot exclude the possibility that other lipids may also contribute to CPT induction in NAFLD.

### NAFLD induces the expression of CPT genes in intrahepatic CD4^+^ T cells

**Fig. 3:**
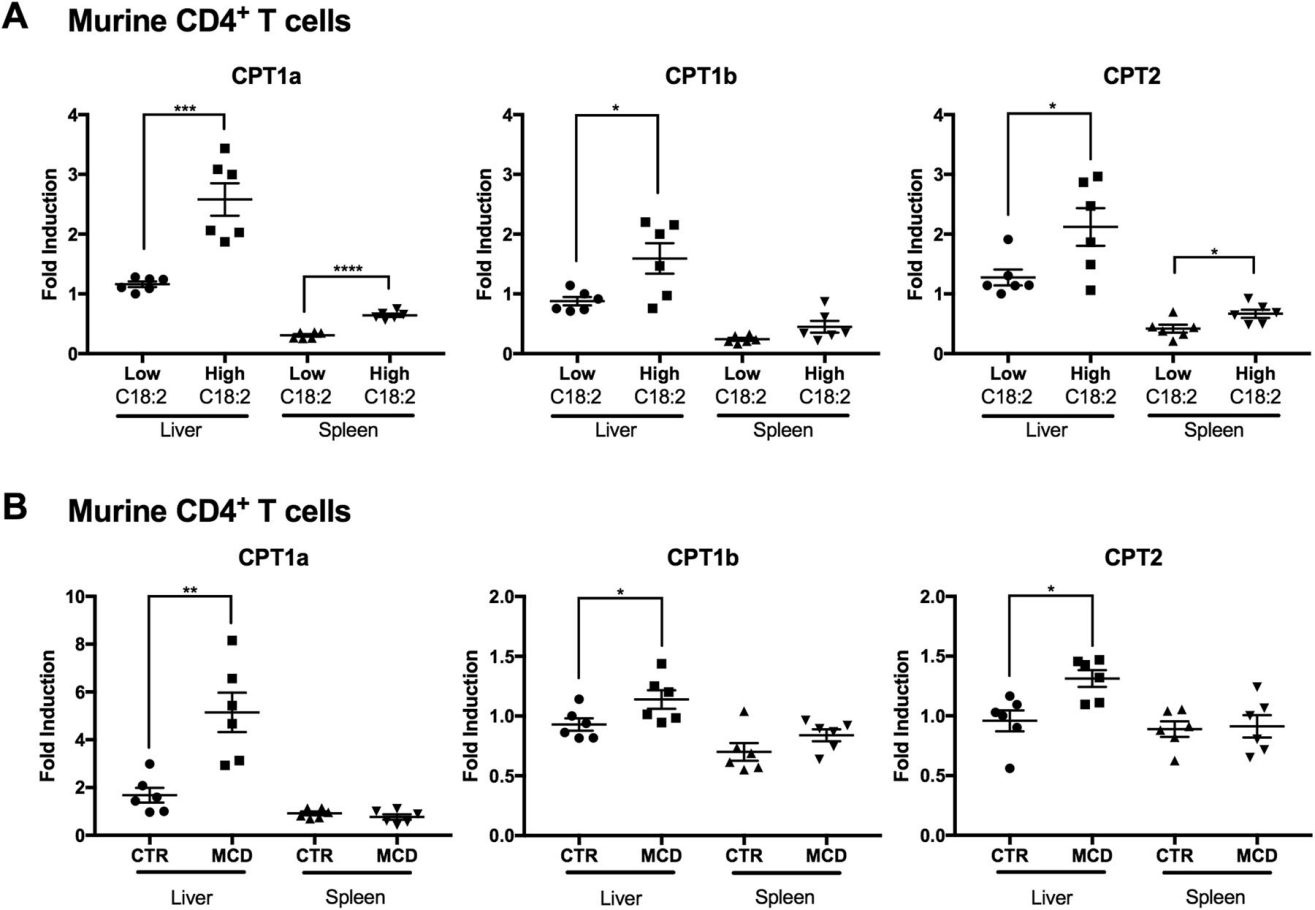
NAFLD induces the expression of CPT genes in intrahepatic CD4^+^ T cells. **a** 8-week-old female C57BL/6 mice were fed with either low C18:2 (2%, w/w) or high C18:2 (12%, w/w) diet for 8 weeks. CD4^+^ T cells were isolated from liver and spleen. CPT levels were measured by RT-qPCR. **b** 8-week-old female C57BL/6 mice were fed with MCD diet or control diet (CTR) for 4 weeks. CD4^+^ T cells were isolated from liver and spleen. CPT levels were measured by RT-qPCR. *P < 0.05, **P < 0.01, ***P < 0.001, ****P < 0.0001

As our previous study demonstrated mice fed with a high-C18:2 diet showed CD4^+^ T cell apoptosis in vivo and we demonstrated C18:2 can upregulate CPT expression in vitro, we hypothesized if mice were fed with a high-C18:2 diet, we would see an upregulation of CPT genes in CD4^+^ T cells^8^. Mice were fed either a high or low C18:2 diet, CD4^+^ T cells were then isolated from the mouse liver or spleen, and mRNA levels of CPT1a, CPT1b, and CPT2 were quantified. A higher expression of CPT1a, CPT1b, and CPT2 was found in CD4^+^ T cells from liver of mice fed the high-C18:2 diet (Fig. 3a). Additionally, an induction of CPT1a and CPT2 was also found in CD4^+^ T cells isolated from the spleen (Fig. 3a).

Subsequently, we sought to test if inducing NAFLD by feeding mice the NAFLD-inducing methionine-choline-deficient (MCD) diet would lead to similar CPT upregulation in CD4^+^ T cells. Figure 3b shows an increase of all three CPT mRNA levels in intrahepatic CD4^+^ T cells after feeding MCD diet. Unlike the high-C18:2 diet which also showed induction of CPT1a and CPT2 in the spleen as well as the liver, induction of CPT genes was not observed in splenic CD4^+^ T cells from MCD diet-fed mice, demonstrating that it was a liver-specific (Fig. 3a, b). High circulating levels of C18:2 in the high-C18:2 diet-fed mice may have caused the induction of CPT genes in the spleen. Of note, the largest induction was seen in CPT1a from CD4^+^ T cells isolated from the liver of mice fed with the MCD diet, indicating there might be other lipids which also contribute to CPT1 induction in NAFLD. These results together suggest that C18:2 is one of the most important fatty acids to induce CPT genes upregulation in the setting of NAFLD.

### CPT gene induction is mediated by PPAR-α

**Fig. 4:**
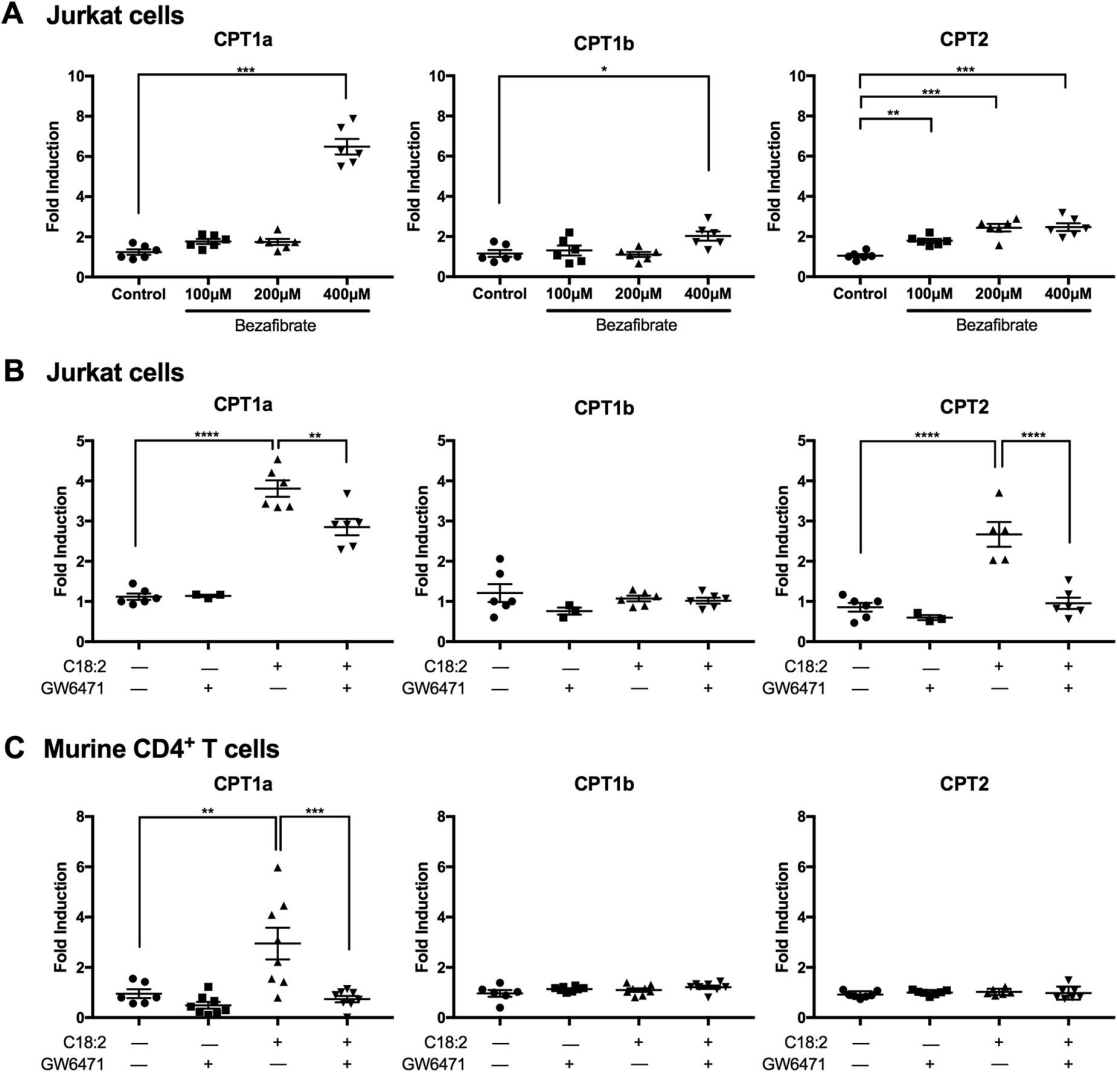
C18:2 upregulates CPT gene expression through PPAR-α. **a** Jurkat cells were cultured with various concentrations of the PPAR-α agonist bezafibrate in vitro for 24 h and CPT mRNA levels were measured by RT-qPCR. **b** Jurkat cells were treated with 200 µM C18:2 in the absence or presence of 1 µg/mL PPAR-α antagonist GW6471 in vitro for 24 h and CPT mRNA levels were measured by RT-qPCR. **c** Mouse splenic CD4^+^ T cells were isolated and treated with 100 µM C18:2 in the absence or presence of 0.25 µg/mL GW6471 in vitro for 24 h. CPT mRNA levels were measured by RT-qPCR. *P < 0.05, **P < 0.01, ***P < 0.001, ****P < 0.0001

As we have demonstrated CPT genes are upregulated in the context of NAFLD, we sought to determine the mechanism behind the CPT upregulation. Peroxisome proliferator-activated receptor alpha (PPAR-α), a transcription factor and a major regulator of lipid metabolism, can be activated by a variety of fatty acids including C18:2^14^. It has also been reported that PPAR-α can directly upregulate CPT1a expression^16, 17^. Therefore, we hypothesized that the induction of CPT genes observed in CD4^+^ T cells after C18:2 treatment is mediated by PPAR-α. To test this, we used the PPAR-α agonist bezafibrate and monitored CPT gene expression. We took advantage of Jurkat cells, which also upregulate CPT genes after C18:2 treatment (Supplementary Fig. 2A). This upregulation of CPT1a mRNA indeed resulted in increased CPT1a protein expression (Supplementary Fig. 2B). A dose-response study demonstrated that the PPAR-α agonist bezafibrate increased the expression of all three CPT genes in Jurkat cells with the greatest induction seen in CPT1a (Fig. 4a). Meanwhile, when the PPAR-α antagonist GW6471 was used to inhibit PPAR-α, the induction of CPT genes caused by C18:2 in Jurkat cells was significantly reduced (Fig. 4b). Similarly, the C18:2-induced CPT1a upregulation seen in murine CD4^+^ T cells was also blocked by GW6471 (Fig. 4c), indicating that PPAR-α mediates the linoleic acid-induced CPT gene expression. Such results were not observed when a PPAR-γ antagonist was used (data not shown). However, as we observed only a partial reduction of CPT1a compared to CPT2 in Jurkat cells, we suspect there may be other contributing factors influencing CPT gene expression.

### CPT upregulation induces apoptosis through ROS

**Fig. 5:**
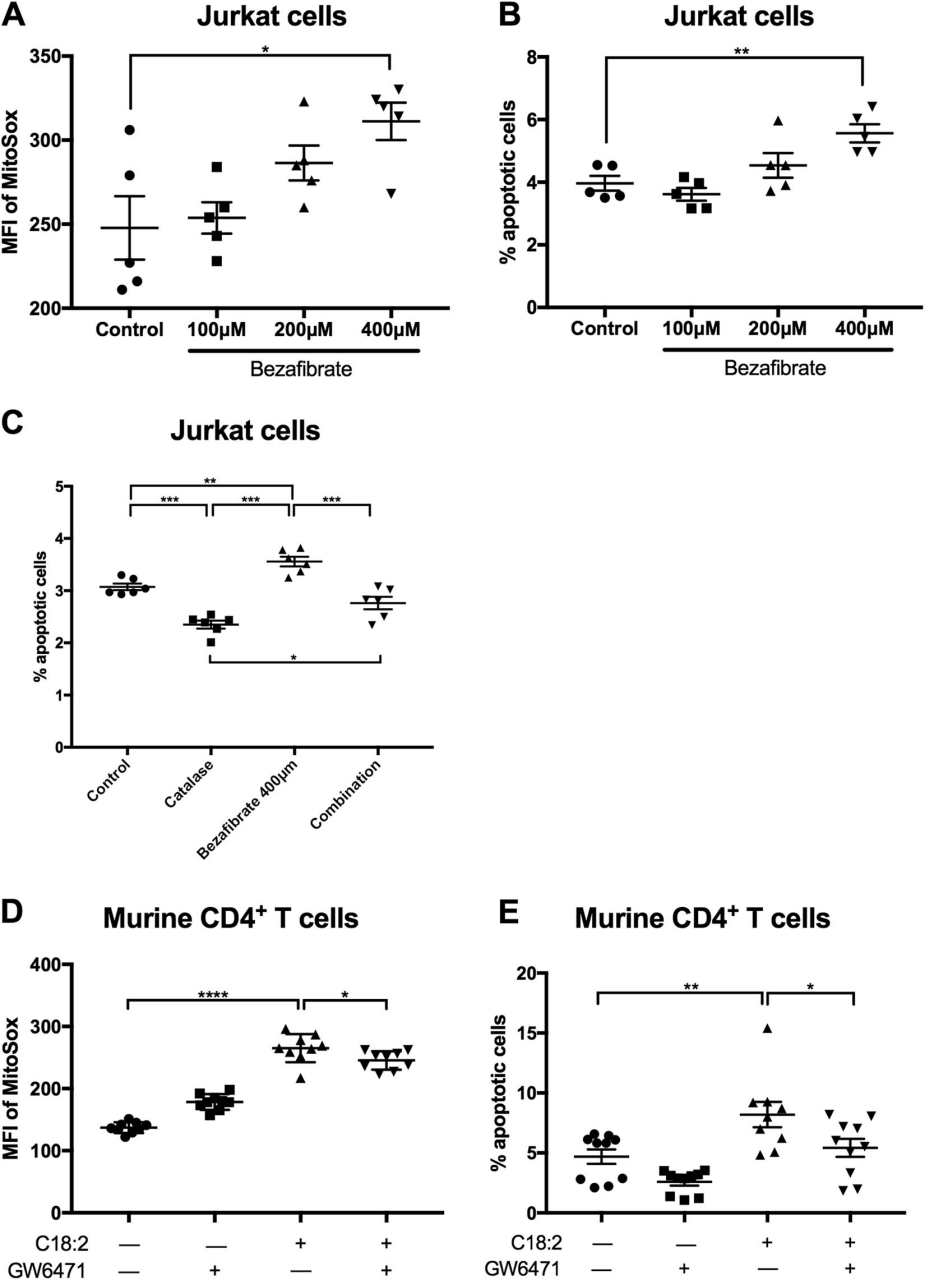
CPT upregulation induces apoptosis through reactive oxygen species. **a** Mitochondrial ROS level was measured by mitoSOX in Jurkat cells treated with various concentrations of the PPAR-α agonist bezafibrate in vitro for 24 h. **b** Jurkat cells were treated with various concentrations of bezafibrate in vitro for 24 h and apoptosis was measured by 7AAD and AnnexinV. **c** Jurkat cells were treated with 400 µM bezafibrate and/or 1000 U/mL catalase in vitro for 24 h and apoptosis was measured by 7AAD and AnnexinV. **d** Murine CD4^+^ T cells were treated with 100 µM C18:2 in the absence or presence of 0.25 µg/mL GW6471 in vitro for 24 h and mitochondrial ROS levels were measured by mitoSOX. **e** Murine CD4^+^ T cells and treated with 100 µM C18:2 in the absence or presence of 0.25 µg/mL GW6471 in vitro for 24 h and apoptosis was measured by 7AAD and AnnexinV. *P < 0.05, **P < 0.01, ***P < 0.001, ****P < 0.0001

We have shown that C18:2 has the greatest induction on CPT1a, and this leads to increased ROS and subsequent CD4^+^ T cell apoptosis^8^. We hypothesized if we augment CPT1a upregulation, we can increase mitochondrial ROS and subsequent apoptosis. Using the PPAR-α agonist, we found increased mitochondrial ROS and corresponding apoptosis in Jurkat cells with a significant increase noted at 400 µM bezafibrate (Fig. 5a, b). This was also observed in murine CD4^+^ T cells treated with bezafibrate (Supplementary Fig. 3A, B). In addition, this apoptosis could be rescued by adding catalase which reduces ROS, indicating the cell apoptosis was indeed the result of ROS production (Fig. 5c). Furthermore, utilizing murine CD4^+^ T cells, we found the PPAR-α antagonist GW6471 was able to reduce ROS and cell apoptosis (Fig. 5d, e), further supporting the importance of PPAR-α in this mechanism.

To rule out the possibility of drug side effect, we utilized stably transfected Jurkat cells where CPT1a is silenced with a construct expressing CPT1a shRNA. We previously demonstrated shRNA silencing of CPT1a rescues cell apoptosis upon C18:2 treatment^8^. Here we further demonstrate that the knockdown cells with greater knockdown efficacy had the lowest ROS production (Supplementary Fig. 3C, D). These results further suggest the role of CPT1a as well as PPAR-α in the metabolism of C18:2, resulting in increased ROS and T cell apoptosis in the context of NAFLD.

### CPT inhibitor perhexiline decreases HCC incidence under NAFLD in vivo

**Fig. 6:**
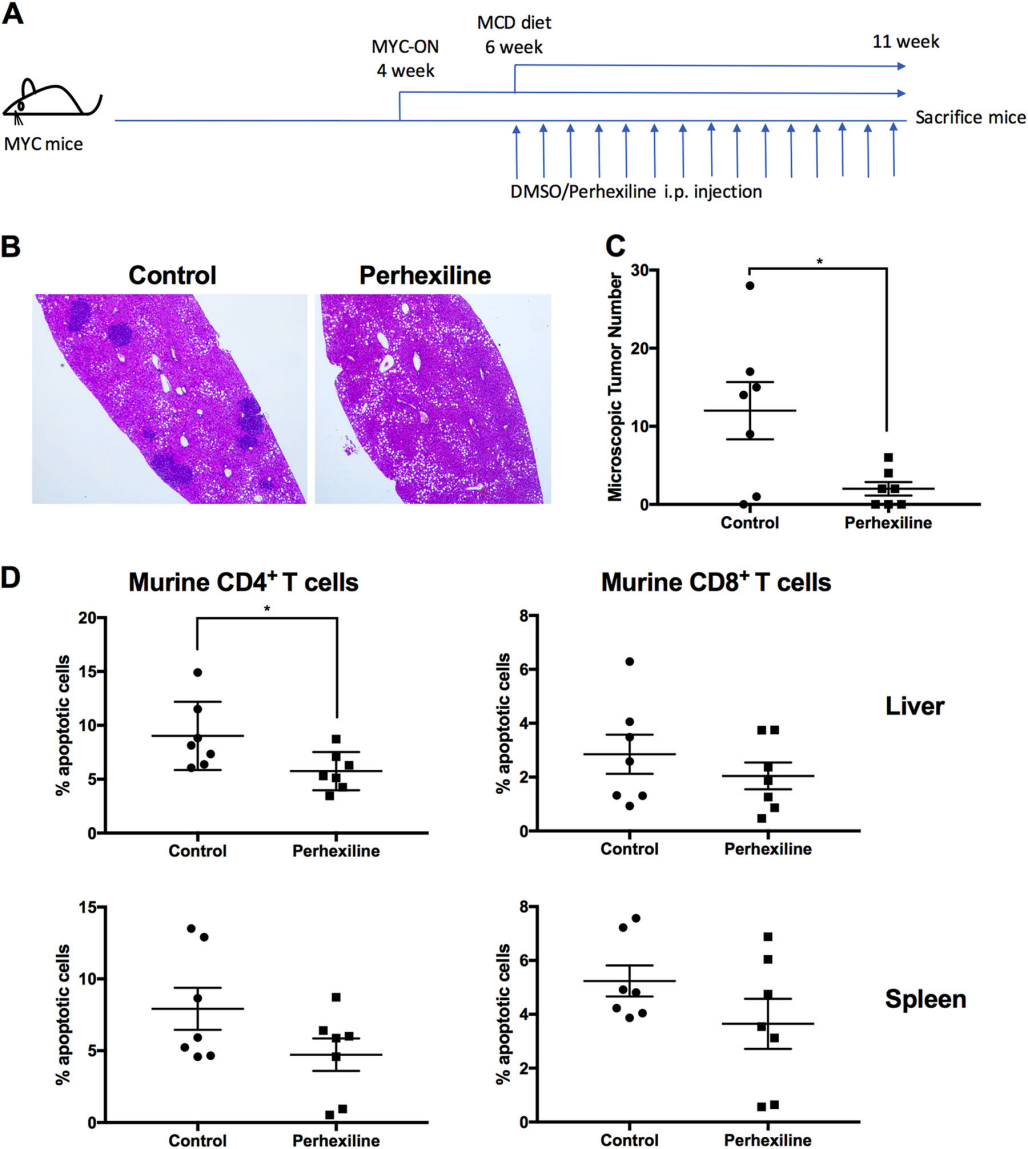
CPT inhibitor perhexiline reduces HCC incidence in MYC-ON mice fed with MCD diet. MYC-ON mice were fed with MCD diet and injected with perhexiline or DMSO control three times per week for 5 weeks. The schematic timeline is shown in **a**. Liver tissue was fixed for H&E staining and the microscopic tumor number was counted. Mice were randomized to each group after doxycycline removed from drinking water (MYC-ON). Representative images of H&E staining (**b**) and microscopic tumor counts (**c**) of fixed liver tissue were shown here. **d** In vivo effect of perhexiline treatment on CD4^+^ T lymphocytes cell apoptosis. *P < 0.05

As CPT1a upregulation leads to greater ROS and CD4^+^ T cell apoptosis, we sought to test whether blocking CPT1 affects CD4^+^ T cells and HCC development in the context of NAFLD. As we found the CPT inhibitor perhexiline rescued murine CD4^+^ T cell and Jurkat cell apoptosis when cultured with C18:2 in vitro (Supplementary Fig. 4A,B), we sought to test if perhexiline could rescue CD4^+^ T cells in vivo and subsequently decrease the incidence of HCC. Liver-specific inducible MYC oncogene transgenic mice, which spontaneously develops HCC after turning on MYC gene (MYC-ON), were fed with the MCD diet and injected with perhexiline. The treatment timeline is shown in Fig. 6a. The perhexiline treatment group showed a significant reduction in the microscopic HCC tumor number (Fig. 6b, c), while it had no effect on liver or spleen weight (Supplementary Fig. 4C). A significant decrease of early apoptotic events in intrahepatic CD4^+^ T cells was observed (Fig. 6d and Supplementary Fig. 4D). This reduction of cell apoptosis was not found in intrahepatic CD8^+^ T cells or CD4^+^ T cells from spleen. These findings indicate that inhibiting CPT with perhexiline can rescue intrahepatic CD4^+^ T cells in vivo and subsequently prevent HCC development in the context of NAFLD.

## Discussion

**Fig. 7:**
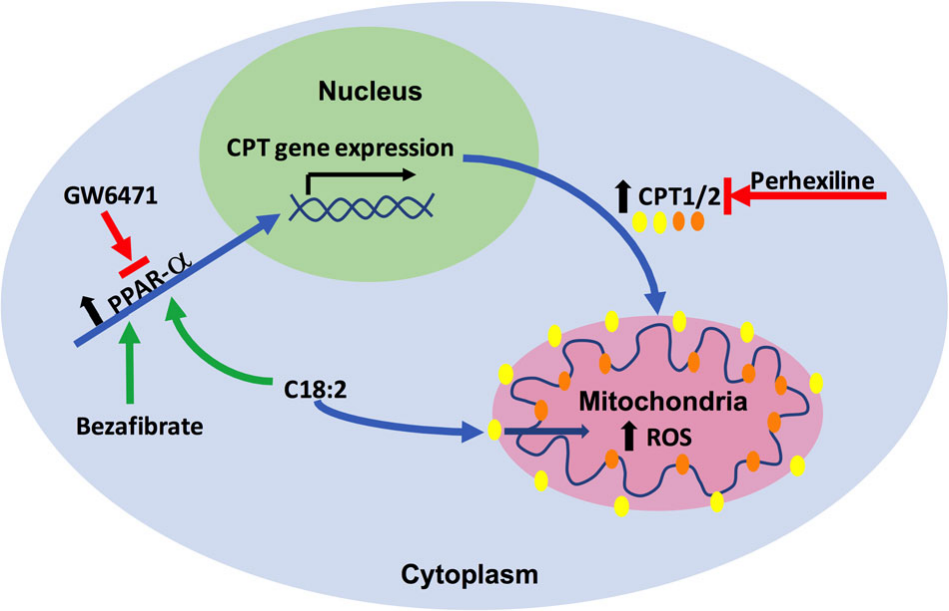
Schematic picture showing CPT genes are upregulated through PPAR-α by C18:2. C18:2 activates the lipid receptor and transcription activator PPAR-α, which translocated to nucleus and upregulates the expression of CPT genes (CPT1 and CPT2). Therefore, increased level of CPT enzymes on mitochondrial membrane promotes the mitochondrial uptake of C18:2, resulting in elevated ROS and cell apoptosis

In this study, we investigated how the fatty liver environment promotes CD4^+^ T cell apoptosis through the upregulation of CPT genes, and if targeting CPT could inhibit the growth of NAFLD-promoted HCC. A liver-selective CD4^+^ T cell induction of CPT1a, CPT1b, and CPT2 was found in NAFLD. In vivo feeding and in vitro culture experiments showed that C18:2 induced CPT gene upregulation. Using either an agonist or antagonist, we provided evidence that PPAR-α mediates the induction of CPT genes. Furthermore, using the CPT inhibitor perhexiline, we demonstrated that in vivo targeting of CPT inhibits HCC development in the context of NAFLD. The molecular mechanism is shown in Fig. 7. Briefly, C18:2 activates the lipid receptor and transcription activator PPAR-α, which translocated to nucleus and upregulates the expression of CPT genes. Therefore, increased level of CPT enzymes on mitochondrial membrane promotes the mitochondrial uptake of C18:2, resulting in elevated ROS and cell apoptosis.

The liver is a central organ for lipid metabolism. With the prevalence of NAFLD in Western countries and the significant risk of NAFLD patients to develop HCC, critical components in lipid metabolism could be potential targets for the treatment of NAFLD-induced HCC. PPARs and CPT proteins are among those potential targets. However, the exact role of PPAR-α in HCC has been controversial on whether PPAR-α promotes or suppresses tumor growth^20^. It has been shown that HCC can arise when PPAR-α agonists are administered to mice or rats for a long period of time^21, 22^. However, another group showed that the PPAR-α knockout mice were more susceptible to DEN-induced HCC at 6 months compared to their wild-type littermates, probably due to PPAR-α’s inhibition on IκBα and NF-κB signaling pathway^23^. These findings are not surprising as PPAR-α is known to regulate a large number of genes, not only in lipid metabolism but also in inflammatory responses, and possibly other pathways as well^24^.

As a result of PPAR-α having a broad influence on multiple genes in different molecular pathways, targeting PPAR-α could have numerous affects which may vary in different settings. Hence, we focused on another set of critical players in lipid metabolism, the CPT genes, hypothesizing that directly targeting CPT genes would reduce HCC incidence. In addition, we chose to target CPT as mitochondria have many critical roles in energy production, as well as cell catabolic processes and several cellular signaling pathways, such as apoptosis. As shown previously, as well as here, CPT family members are the central mitochondrial membrane proteins that regulate the uptake of long-chain fatty acids which lead to hepatic CD4^+^ T cell apoptosis and promote HCC. Collectively, these highlight CPT as an ideal target for NAFLD-promoted HCC therapy.

Perhexiline was previously utilized for the treatment of angina pectoris, and later it was found to act as a cardiac metabolic agent inhibiting the activity of CPT1 and to a lesser extent CPT2^25^. The use of perhexiline in cardiac disease has decreased significantly due to hepatic and neurologic adverse events^26^. In our mouse model utilizing perhexiline, we did not observe any obvious adverse side effects between the treatment group and the control group. Inhibition of CPT via perhexiline has been studied in other cancers as well. Liu et al. observed positive results utilizing perhexiline in a mouse model of chronic lymphocytic leukemia as it has been observed there is a deregulation of lipid metabolism in leukemia cells^27, 28^. Additionally, etomoxir, another CPT inhibitor, has been explored in treatment of acute myeloid leukemia^29^. We did not use etomoxir for our in vivo HCC study because we could not observe a rescue of apoptosis in Jurkat cells by etomoxir when treated with C18:2 in vitro (data not shown). Furthermore, Pucci et al. demonstrated knockdown of CPT1a by siRNAs was able to induce apoptosis in breast cancer cell lines^30^. No study targeting CPT has been performed in the context of HCC or HCC cell lines. In this study, we also noted a difference in CPT expression between CD4^+^ T cells and Jurkat cells under different experimental conditions. This is not a surprise as Jurkat cells are a human T lymphocyte cell line and altered fatty acid metabolism has been described in cancer cells^31^.

In addition to upregulation of CPT1a, we also noted an induction of CPT2 in human CD4^+^ T cells and Jurkat cells when cultured in vitro with C18:2 as well as in murine CD4^+^ T cells when mice were fed either C18:2 high or MCD diet. CPT1 is the rate limiting step for the metabolism of fatty acids as it catalyzes the first reaction to shuttle fatty acids into the mitochondria^13, 32^. Inhibiting CPT1 would result in the interruption of the fatty acid transportation into mitochondria therefore blocking the whole process of long-chain fatty acid metabolism.

Previously it has been shown that alterations in T cell metabolism can influence and produce a suppressive immune phenotype where T cell metabolism to influence immune function. For example, the immunosuppressive molecule indoleamine 2,3-dioxygenase (IDO), the first enzyme in tryptophan metabolism, has been implicated in promoting immune-inhibitory effects through increasing regulatory T cell differentiation, altering natural killer and dendritic cell function, and well as the direct inhibitory effects of the tryptophan byproduct kynurenine^33, 34^. Eleftheriadis et al. demonstrated in the absence of IDO inhibition, there is increased fatty acid metabolism in T cells with increased CPT1 expression which was associated with increased differentiation into regulatory T cells^35^. Additionally, the increase in CPT1 expression may also produce an immunosuppressive microenvironment through increasing CD4^+^ T cell apoptosis in the setting of fatty liver disease as we have shown. Therefore, targeting CPT family members might also help to overcome the immunosuppressive environment in NAFLD-induced HCC, and potentially be developed into a combinational immunotherapy.

In this study, we have focused on CPT and PPAR-α in the context of fatty acid metabolism in T cells. Our prior study has shown that because CD4^+^ T cells have greater mitochondrial mass than CD8^+^ T cells, C18:2 causes increase in CPT1a with impairment of the ETC, production of ROS, subsequent CD4^+^ T cell apoptosis, and the development of HCC^8^. Here, we have further demonstrated pharmacological inhibition of CPT1 can reduce CD4^+^ T cell apoptosis and protect again HCC in the setting of NAFLD. PPAR-α and CPT proteins are not specific to T cells and oxidative stress is also an important factor in NAFLD progression where increased ROS and decreased antioxidant compounds have been found in patients with NAFLD/NASH compared to healthy patients^36, 37^. Additionally, further studies on how hepatocytes and other immune cells respond to CPT inhibition and thus contribute to HCC development are still needed.

To summarize, we demonstrated that by inhibiting CPT1 we can rescue CD4^+^ T cells and prevent HCC development in transgenic mice with an inducible liver-specific MYC oncogene. Our results provide useful information that CPT1 may be a potential target for NAFLD-promoted HCC therapy.

## Materials and methods

### Cell lines

Jurkat cells, a human T lymphocyte-derived cell line, were purchased from the German Collection of Microorganisms and Cell Cultures (DSMZ). Cells were cultured in complete Roswell Park Memorial Institute (RPMI) medium supplemented with 10% fetal bovine serum (Gibco). 3T3 Swiss albino fibroblast cells were purchased from American Type Culture Collection and cultured in Dulbecco’s Modified Eagle’s Medium supplemented with 10% fetal bovine serum. Cells from early passages were used for all described experiments^8^. No authentication test was performed by our lab. Mycoplasma tests were negative for all cell lines used in this study.

### Mouse studies

C57BL/6 mice were purchased from Charles River Laboratories (VA, USA) at 8–10 weeks of age. NAFLD was induced by feeding mice the MCD diet (catalog number 960439, MP Biomedicals), or custom made high C18:2 (12%) or low C18:2 (2%) diet (Research Diets) as previously described^8^. Liver-specific inducible MYC oncogene transgenic mice (MYC-ON) have been previously used by our group where MYC expression in the liver was activated by removing doxycycline treatment from the drinking water of 4-week-old double transgenic mice for TRE-MYC and LAP-tTA^8, 38, 39^. The CPT inhibitor, perhexiline maleate (Cayman Chemical, MI, USA) was given intraperitoneally to MYC-ON mice at 8 mg/kg in 100 µL 50/50 solution of PBS/DMSO three times per week from week 6 to week 11, during which time the mice were fed with the MCD diet^27^. MYC-ON mice were injected i.p. with 100 µL of vehicle without perhexiline as control. Mice were approximately 20 g at time of injections. Upon removal of the liver, sections of the liver were fixed with 10% formaldehyde and sent to Histoserv (Germantown, MD, USA) for hematoxylin and eosin (H&E) staining. All animal experiments were performed according to the institutional guidelines and approved by a NCI-Bethesda (Bethesda, MD, USA) Institutional Animal Care and Use protocol.

### Human studies

Human blood samples were drawn from health volunteers after obtaining informed consent for peripheral blood mononuclear cell (PBMC) isolation and prepared as previously described^40^. Briefly, fresh PBMCs were isolated by Ficoll density gradient centrifugation (Biochrom, Berlin, Germany). CD4^+^ or CD8^+^ T cells were isolated from the lymphocyte preparations via the autoMACS (Miltenyi Biotec) with negative selection utilizing either a human CD4^+^ or human CD8^+^ T lymphocyte isolation kit (Miltenyi Biotec). One million human CD4^+^ or CD8^+^ T cells were then cultured in 200 µL RPMI medium in a 96-well plate with 100 µM of C18:2 or C16:0 for 24 h.

### Drugs

The peroxisome PPAR-α agonist bezafibrate (Cayman Chemical, MI, USA), the PPAR-α antagonist GW6471 (Cayman Chemical, MI, USA), and/or the CPT1 inhibitor perhexiline maleate (Cayman Chemical, MI, USA) were cultured with Jurkat cell in vitro at the indicated concentrations^41, 42^. Catalase (Sigma-Aldrich) was used to inhibit ROS production at 1000 U/mL^8^. Perhexiline maleate was injected intraperitoneal (i.p.) to inducible liver-specific MYC oncogene transgenic mice (MYC-ON) at 8 mg/kg in 100 µL 50/50 solution of PBS/DMSO three times per week for a total of 5 weeks^27^.

### CD4^+^ T cell isolation and co-culture with fatty acids

Single-cell suspensions of lymphocytes were prepared from the liver and spleen of C57BL/6 mice. Red blood cell lysis was performed with ACK Lysis Buffer (Quality Biologicals, MD, USA). CD4^+^ or CD8^+^ T cells were isolated from the lymphocyte preparations via the autoMACS pro with negative selection utilizing either a mouse CD4^+^ or a mouse CD8^+^ T lymphocyte isolation kit (Miltenyi Biotec)^8^. One million T lymphocytes were then cultured in 200 µL RPMI medium in a 96-well plate with 100 µM of linoleic acid (C18:2) or palmitic acid (C16:0) (Sigma-Aldrich) for 24 h^8^. One hundred thousand Jurkat cells were cultured with various concentrations of C18:2 in 200 µL RPMI medium in a 96-well plate for 24 or 48 h.

### RNA isolation and real-time PCR

Total RNA was extracted from cell pellets or frozen tissue with QIAshredder (Qiagen) and RNeasy MiniKit (Qiagen). Complementary DNA was synthesized by iScript^TM^ cDNA synthesis kit (Bio-Rad). The sequence of primers used for quantitative RT-PCR can be found in Supplementary Tables 1 and 2. All reactions of quantitative RT-PCR were run in triplicates using iQ^TM^ SYBR Green Supermix (Bio-Rad) and performed on the ViiA^TM^ 7 Real-Time PCR System (Life Technologies). The results were normalized to endogenous GAPDH expression levels first and then reported as fold induction (2-ΔΔCt)^8^.

### Mitochondrial ROS staining

Mitochondrial-associated ROS was detected by mitoSOX (Life Technologies) staining according to the manufacturer’s protocol. Briefly, treated cells were stained with 2.5 µM mitoSOX for 30 min in a CO_2_ incubator at 37 °C. After washing twice, the cells were processed by flow cytometry analysis as below^8^.

### Flow cytometry analysis

Cells were surface labeled with the indicated antibodies at 4 ˚C for 15 min. The following antibodies were used for flow cytometry: anti-CD3-APC-Cy7 (clone 145-2C11, BioLegend), anti-CD4-AF700 (clone GK1.5, BioLegend), anti-CD8-PB (clone 53–6.7, BioLegend), and cell death-7-AAD (BD Pharmingen). Apoptosis was detected with Annexin V-PE (BD Pharmingen) staining according to the manufacturer’s instructions^8^. Gating strategy is outlined in Supplementary Fig. 5. The percentage of apoptotic Jurkat cells was obtained by utilizing the PE Annexin V Apoptosis Detection Kit (BD Pharmingen) according to the manufacturer’s instructions. Flow cytometry was performed on BD Fortessa or Beckman-Coulter CytoFLEX LX platforms. Results were analyzed using FlowJo software version 10.0.8r1 (TreeStar).

### CPT gene knockdown by shRNA

Three of each pZip-TRE3G lentiviral shRNA vectors targeting human CPT1a and a control vector (NT#4: non-targeting: #4) were purchased from TransOMIC Technologies. The sequence is shown in Supplementary Table 3. This method has been previously described^8^. Briefly, lentivirus was packed in 293T cells and harvested to infect Jurkat cells. After infection, puromycin was added to select for transduced cells. Doxycycline (100 ng/mL) was also added to induce shRNA and GFP expression for 3 days. After knockdown, Jurkat cells were treated with or without 200 µM C18:2 for 24 h and then mitochondrial ROS levels were detected by mitoSOX.

### Western blotting

CPT shRNA knockdown Jurkat cells were lysed with 1× Laemmli Sample Buffer (Bio-Rad, #1610747) and the whole-cell lysates were fractionated by 4–20% SDS-PAGE (MINI-PROTEAN TGX, 4–20%, Bio-Rad) and transferred to a PVDF membrane. The membrane was incubated with antibodies against CPT1a (1 µg/ml, ab128568, Abcam) and GAPDH (1:2000, SC-25778, Santa Cruz) at 4 °C overnight, and subsequently incubated with 1:5000 dilution of horseradish peroxidase-conjugated goat anti-rabbit or anti-mouse antibodies (Jackson ImmunoResearch) for 1 h at room temperature. The relative CPT expression level was determined by comparing to the control shRNA (NT#4) after first normalizing to GAPDH level.

For CPT1a protein expression, Jurkat cells were incubated with or without 200 µM C18:2 for 24 or 48 h, as indicated. The harvested cells were then lysed and the whole-cell lysates were subjected for western blot with antibodies against CPT1a (1 µg/ml, ab83862, Abcam) and GAPDH (1:2000, SC-343 25778, Santa Cruz) as described above.

### Synthesis of linoleic acid probes

A general synthesis of Z,Z-bodipy substituted dienes has been developed here. This new approach uses a selective hydrogenation of the corresponding diyne in the presence of the Bodipy fluorescent group. Synthesis of linoleic acid probe 1 (LAP1) is depicted in scheme 1 (Supplementary Fig. 6A). Ethynyltrimethylsilane 1 was alkylated with Boc-protected 3-iodopropyl-pyrrole followed by deprotection with sodium methoxide to produce compound 3. The pyrrole ring of compound 3 was further substituted with 5-methyl-1H-pyrrole-2-carbaldehyde and mixed with BF_3_•Et_2_O to form the Bodipy compound 4^43^. Ethyl 11-hydroxyundec-9-ynoate was brominated and coupled with compound 4 in the presence of copper (I) catalyst and cesium carbonate to provide the fluorescent diyne product 7. The two alkynes group of compound 7 were hydrogenated to Z,Z-diene 8 using Lindlar’s catalyst^44, 45^. Subsequently, compound 8 was hydrolyzed under aqueous hydrochloride acid to produce the final probe LAP1.

Based on the synthetic route above, linoleic acid probe 2 (LAP2) was prepared as shown in scheme 2 (Supplementary Fig. 6B). Tert-butyl 3-(5-formyl-1H-pyrrol-2-yl) propanoate 9 was substituted with pyrrole 3 and mixed with BF_3_•Et_2_O to provide BODIPY 10. Compound 10 was coupled with 1-bromooct-2-yne to yield the diyne Bodipy 11. After hydrogenation with Lindlar’s catalyst and hydrolysis, the final probe LAP2 was obtained.

### Fluorescent staining and confocal microscopy

3T3 Swiss fibroblast cells were seeded into 8-well Nunc^TM^ Lab-Tek^TM^ Chambered Coverglass wells (Thermo Fisher) at a concentration of 10^5^ cells per well. After 16 h, cells were washed with phosphate-buffered saline (PBS) three times and incubated with 2 µM C18:2 BODIPY probe 1 or 2 (described as above) with 10 nM MitoTracker Deep Red (Thermo Fisher) in PBS at 37 ˚C for 30 min. Cells were again washed with PBS and kept in DPBS (with calcium, magnesium; Thermo Fisher) for imaging. Live cells were imaged using a Zeiss LSM880 laser scanning confocal microscope equipped with a 32-channel GaAsP spectral detector, a 63× plan-aprochromat (N.A. 1.4) objective lens, and a stage-top incubator for control of temperature, CO_2_, and humidity. Images were collected at 1 or 24 h after staining.

Jurkat cells were incubated with PBS containing 2 μM C18:2-BODIPY Probe 1 or Probe 2 and 20 nM MitoTracker Deep Red for 30 min then washed with PBS for three times. After that, cells were applied to poly-L-lysine pre-coated 8-well NuncTM Lab-TekTM Chambered 366 Coverglass wells (Thermo Fisher) at a concentration of 10^6^ cells per well. Live images were taken 1, 6, or 24 h after staining.

### Statistical analysis

Sample sizes for animal studies were guided by previous studies in our laboratory in which the same C57BL/6 and MYC-ON mouse strains were used. For the MYC-ON mice, the same littermates were randomly distributed into control or treatment group when possible. Statistical analysis was performed using the GraphPad Prism v7.03 (GraphPad Software). Significance of the difference between groups was calculated by Student’s unpaired *t*-test, one-way or two-way ANOVA (Tukey’s and Bonferroni’s multiple comparison test). Welch’s corrections were used when variances between groups were unequal. *P* < 0.05 was considered as statistically significant^8^.

## Electronic supplementary material

Supplemental Data: Clean
