# Supplementary material for: Carnitine palmitoyltransferase gene upregulation by linoleic acid induces CD4+ T cell apoptosis promoting HCC development
Source: Cell Death Dis. 2018 May 23;9(6):620. doi: 10.1038/s41419-018-0687-6 (PMC5966464; doi:10.1038/s41419-018-0687-6)
Supplement: Supplementary file 1 — Supplemental Data: Clean [file 41419_2018_687_MOESM1_ESM.docx]

**Supplemental Data:**

**Carnitine palmitoyltransferase gene upregulation by linoleic acid induces CD4^+^ T cell apoptosis promoting HCC development.**

Zachary J. Brown, Qiong Fu, Chi Ma, Michael Kruhlak, Haibo Zhang, Ji Luo, Bernd Heinrich, Su Jong Yu, Qianfei Zhang, Andrew Wilson, Zhen-Dan Shi, Rolf Swenson, Tim F. Greten.

**Table of Contents:**

**Supplementary Figures 1-6: p 2-11**

**Supplementary Tables 1-3: p 12-13**

**Supplementary Figure 1: Linoleic acid probes co-localize with the mitochondria of Jurkat Cells.**

Jurkat cells were incubated with 2µM C18:2-BODIPY Probe 1 or Probe 2 together with 20nM MitoTracker Deep Red for 30 mins. Live images were taken at 1h, 6h, or 24h after staining. Scale bar: 20μm.

**Supplementary Figure 2: Jurkat cells upregulate CPT genes after C18:2 treatment.**

1. Jurkat cells cultured *in vitro* with various concentrations of C18:2 demonstrated CPT induction as measured by RT-qPCR.
2. Jurkat cells were incubated with or without 200µM C18:2 for 24h or 48h. The harvested cells were then lysed and the whole cell lysate was subjected for western blot with antibodies against CPT1a and GAPDH.

*: P<0.05, ***: P<0.001

**Supplementary Figure 3: CPT genes induce apoptosis through ROS.**

1. Mitochondrial ROS level was measured by mitoSOX in murine CD4^+^ T cells treated with various concentrations of the PPAR-α agonist bezafibrate *in vitro* for 24 hours.
2. Murine CD4^+^ T cells were treated with various concentrations of bezafibrate *in vitro* for 24 hours and apoptosis was measured by 7AAD and AnnexinV.
3. Expression level of CPT1 in non-targeting (NT) or CPT1 knocked-down Jurkat cell lines was detected by western blotting.
4. Mitochondrial ROS level was measured by mitoSOX in wild-type (NT: non-targeting) or CPT1 knockdown GFP^+^ Jurkat cells treated with 200µM C18:2 *in vitro* for 24 hours.

****: P<0.0001

**Supplementary Figure 4: Perhexiline rescues cell apoptosis in context of NAFLD.**

1. Murine CD4^+^ T cells were treated with 100µM C18:2 and/or 0.3125µM perhexiline for 48 hours *in vitro* and cell apoptosis was measured by 7AAD and AnnexinV.
2. Jurkat cells were treated with 200µM C18:2 and/or 0.625µM perhexiline for 48 hours *in vitro* and cell apoptosis was measured by 7AAD and AnnexinV.
3. Liver and Spleen weight of sacrificed MYC-ON mice which were fed with MCD diet and injected with perhexiline or DMSO control 3 times per week for a total of 5 weeks.
4. *In vivo* effect of perhexiline treatment on the number of apoptotic CD4^+^ or CD8^+^ T lymphocytes per gram liver tissue.

*: P<0.05, ****: P<0.0001

**Supplementary Figure 5: Gating strategy for early apoptotic cells**

Lymphocytes gate drawn followed by Live Dead staining using 7-AAD against forward scatter. On live cells, double positive CD3^+^ and CD4^+^ T cells as well as CD3^+^ and CD8^+^ T cells were gated upon which early apoptosis was determined by AnnexinV staining.

**Supplementary Figure 6: Synthesis of linoleic acid probes**

A. Scheme 1: synthetic approach of linoleic acid probe 1 (LAP1).

B. Scheme 2: synthetic approach of linoleic acid probe 2 (LAP2).

**Supplementary Table 1: Mouse Primers (5’🡪3’)**

| CPT1a Forward | CTCAGTGGGAGCGACTCTTCA |
| --- | --- |
| CPT1a Reverse | GGCCTCTGTGGTACACGACAA |
| CPT1b Forward | ACTGAGACTGTGCGTTCCTG |
| CPT1b Reverse | GTGCTTTTCGGAGGCTTTCC |
| CPT2 Forward | CCCTGCATACCAGCGGATAA |
| CPT2 Reverse | CATACGCAATGCCAAAGCCA |
| GAPDH Forward | CCTGCACCACCAACTGCTTA |
| GAPDH Reverse | TCATGAGCCCTTCCACAATG |

**Supplementary Table 2: Human Primers (5’🡪3’)**

| CPT1a Forward | CCAGACGAAGAACGTGGTCA |
| --- | --- |
| CPT1a Reverse | ATCTTGCCGTGCTCAGTGAA |
| CPT1b Forward | CTGGGCTATGTGTATCCGCC |
| CPT1b Reverse | GCACAGACTCTAGGTACCGC |
| CPT2 Forward | GTAGCACTGCCGCATTCAAG |
| CPT2 Reverse | GCCATGGTACTTGGAGCACT |
| GAPDH Forward | GAAGGTGAAGGTCGGAGTC |
| GAPDH Reverse | GAAGATGGTGATGGGATTTC |

**Supplementary Table 3: Human shRNA sequence (5’🡪3’)**

| CPT1a#1 | TGCTGTTGACAGTGAGCGCCTGGACCGGGAGGAAATCAAATAGTGAAGCCACAGATGTATTTGATTTCCTCCCGGTCCAGTTGCCTACTGCCTCGGA |
| --- | --- |
| CPT1a#2 | TGCTGTTGACAGTGAGCGCAGGAATAATTGCAAAAATCAATAGTGAAGCCACAGATGTATTGATTTTTGCAATTATTCCTATGCCTACTGCCTCGGA |
| CPT1a#3 | TGCTGTTGACAGTGAGCGCCCTTAAGGAAGTTTTATCTGATAGTGAAGCCACAGATGTATCAGATAAAACTTCCTTAAGGATGCCTACTGCCTCGGA |
| NT#4 | TGCTGTTGACAGTGAGCGAAGGCAGAAGTATGCAAAGCATTAGTGAAGCCACAGATGTAATGCTTTGCATACTTCTGCCTGTGCCTACTGCCTCGGA |
